# Modeling APC mutagenesis and familial adenomatous polyposis using human iPS cells

**DOI:** 10.1371/journal.pone.0200657

**Published:** 2018-07-19

**Authors:** Cesar A. Sommer, Amalia Capilla, Francisco J. Molina-Estevez, Andreia Gianotti-Sommer, Nicholas Skvir, Ignacio Caballero, Sanjib Chowdhury, Gustavo Mostoslavsky

**Affiliations:** 1 Section of Gastroenterology, Department of Medicine, Boston University School of Medicine, Boston, Massachusetts, United States of America; 2 Center for Regenerative Medicine (CReM), Boston University School of Medicine, Boston, Massachusetts, United States of America; Faculty of Biochemistry, Biophysics and Biotechnology, Jagiellonian University, POLAND

## Abstract

Mutations in the gene Adenomatous Polyposis Coli or APC appear in most sporadic cases of colorectal cancer and it is the most frequent mutation causing hereditary Familial Adenomatous Polyposis. The detailed molecular mechanism by which APC mutations predispose to the development of colorectal cancer is not completely understood. This is in part due to the lack of accessibility to appropriate models that recapitulate the early events associated with APC mediated intestinal transformation. We have established a novel platform utilizing human induced Pluripotent Stem cells or iPSC from normal or FAP-specific APC mutant individuals and evaluated the effect of the mutation in the cells before and after differentiation into intestinal organoids. In order to minimize genetic background effects, we also established an isogenic platform using TALEN-mediated gene editing. Comparison of normal and APC mutant iPSC revealed a significant defect in cell identity and polarity due to the presence of APC in heterozygosity as well as chromosomal aberrations including abnormal anaphases and centrosome numbers. Importantly, upon specification into intestinal progeny, APC heterozygosity was responsible for a major change in the transcriptional identity of the cells with dysregulation of key signaling pathways, including metabolic reprogramming, abnormal lipid metabolism and intestinal-specific cadherin expression. In conclusion, we have developed a novel iPSC/intestinal model of APC mutagenesis and provide strong evidence that APC in heterozygosity imparts a clear phenotypic and molecular defect, affecting basic cellular functions and integrity, providing novel insights in the earlier events of APC-mediated tumorigenesis.

## Introduction

Colorectal cancer (CRC) is the second leading cause of cancer-related death in America. Ten to 30% of CRC have a major hereditary component providing unique opportunities to study specific genes and pathways associated with intestinal tumorigenesis. Among them, one of the most important drivers of CRC is Adenomatous Polyposis Coli (APC) as more than 50% of hypermutated tumors and more than 80% of non-hypermutated tumors have mutations in APC [1, 2]. Furthermore, loss of heterozygosity (LOH) of APC has been shown to be one of the earliest and rate-limiting events in the progression from normal epithelium to adenoma formation and then cancer [3–5]. One of the first described functions of APC was to be part of the destruction complex (together with GSK3 and AXIN) that mediates phosphorylation of β-catenin and its ubiquitination and degradation. For years, the field accepted the causal relationship between mutated APC, dysregulated Wnt signaling, proliferation and CRC development. However, improved diagnostic tools and better access to early lesions have found that this is not the case, at least during the earliest stages of malignant transformation [6–8], prompting investigators to study other known functions of APC and their potential role in the initiation of intestinal cancer [9]. In this regard, it is still under debate whether APC in heterozygosity can already alter the molecular and cellular phenotype of the affected cells providing a fertile environment for the initiation of tumorigenesis [10, 11].

We sought to develop a novel approach based on the generation of induced pluripotent stem cells (iPSC) from normal individuals or from patients with familial adenomatous polyposis (FAP), the second most highly penetrant form of hereditary CRC, which is caused mainly by APC mutations. The ability of these pluripotent cells to differentiate into intestinal organoids provided us with a readily accessible platform in which to evaluate the effect of APC mutations on the cellular and molecular phenotype of iPSC and their intestinal progeny. Indeed, by comparing isogenic cells expressing either the wild-type or a truncated version of APC, mimicking the germline mutation commonly found in FAP patients, we detected surprising defects in cell identity and chromosomal integrity. Importantly, upon specification into intestinal progeny, APC heterozygosity was responsible for a major change in the transcriptional identity of the cells with dysregulation of key signaling pathways, including metabolic reprogramming, abnormal lipid metabolism and cadherin overexpression, providing novel insights in the earlier events of APC-mediated tumorigenesis.

## Materials and methods

### iPSC derivation and culture

Reprogramming of fibroblasts from normal and FAP patients was performed as previously described using the excisable STEMCCA lentiviral reprogramming system [12]. Twenty-five to thirty days post-transduction, iPSC colonies were mechanically isolated and expanded on inactivated mouse embryonic fibroblasts (MEFs) in iPSC media, consisting of: DMEM/F12 containing 20% KnockOut Serum Replacement (Invitrogen), 1 mM L-glutamine (Invitrogen), 0.1 mM b-mercaptoethanol (Sigma-Aldrich), 1% nonessential amino acid solution (Invitrogen), and 10 ng/ml of FGF2 (Invitrogen). All human samples collected at the CReM were processed and handled according to the procotol # H-29791 approved by the Institutional Review Board (IRB) Committee of Boston University. The FAP fibroblasts were obtained from the Coriell Institute for Medical Research (GM03948; GM03954; GM06888; GM06965)

Pluripotency marker expression of reprogrammed iPSC was tested using the ES Characterization Kit (Millipore # SCR001) by following manufacture’s recommendations for AP, TRA-1-80, and SSEA4 staining. Colonies were analyzed with the inverted microscope Nikon Eclipse TS100. Karyotype analysis was done by Cell Line Genetics.

### Design and assembly of vectors encoding transcription activator-like effector nucleases (TALENs)

Targeting of the human APC locus in iPSC was achieved by transient delivery of plasmid vectors encoding TALENs. TALEN arrays specific for the APC gene were designed using the TAL Effector Nucleotide Targeter 2.0 software, available at https://tale-nt.cac.cornell.edu. We selected six combinations of TALEN pairs that bind close to the sequence encoding amino acid 1309 of the APC protein. The APC^1309^ mutation is one of the most common APC germline mutations leading to severe intestinal phenotypes. Custom TALEN arrays were assembled as described[13] using the Golden Gate TALEN kit, a gift from Daniel Voytas and Adam Bogdanove (Addgene kit # 1000000024). These TALEN arrays were then subcloned into the pHAGE2 EF1α vector for high-level expression in mammalian cells. Different TALEN combinations were first tested in HEK293 cells and the TALEN pair resulting in the highest frequency of non-homologous end joining (NHEJ) events, as determined by Surveyor Nuclease assay (Integrated DNA technologies), was selected for further experiments.

### Targeting of the APC locus in human iPSC

In order to target the APC gene in iPSC, APC TALENs 1320-L and 1320-R3 were subcloned into the pHAGE2 EF1α IRES Puro vector for co-expression of a TALEN monomer and the Puro resistance gene. We reasoned that transient puromycin selection of iPSC expressing high levels of the TALENs and the *Puro* gene would increase the probability to pick colonies carrying frameshift mutations resulting in premature stop codons. To achieve this, we followed a transfection/selection protocol that we had developed previously for the excision of lentiviral cassettes from the genome of iPSC [12]. In brief, iPSC were cultured until 30% confluent and transfected with the TALEN-Puro vectors using the Hela Monster transfection reagent (Mirus) according to manufacturer’s instructions. The following day, the media was removed and iPSC media containing 1.2 μg/mL puromycin was added. Selection was kept for 48 hours. iPSC colonies re-emerged within 1 week and were picked and expanded as described[12]. Clone screening was performed by PCR amplification of a 612 bp region of genomic DNA surrounding codon 1309. Candidate clones were further interrogated by DNA sequencing.

### Western blot analysis

Protein extraction and Western blot was performed as described elsewhere[14]. For APC detection, 100 μg of protein was loaded on each lane and blotted membranes were stained with specific antibodies for the N-terminus or C-terminus of APC (S1 Table).

### Fibroblast differentiation and scratch assay

In vitro differentiation of iPSC into fibroblasts was done as follows. iPSC were harvested and allowed to form embryoid bodies (EBs) in DMEM media containing 20% KSR. Seven days later, EBs were transferred to 10-cm gelatin-coated culture dishes containing DMEM media with 10% fetal calf serum (FCS). 50–60% confluency cultures were maintained in absence of bFGF and Matrigel and split weekly with trypsin-EDTA for at least 5 passages until achieving uniform fibroblast-like morphology, loss of Tra-1-81, expression of CD90 and normalized G0-G1 cell cycle accumulation. Confluent iPSC-derived fibroblasts were scratched with a pipette tip and allowed to polarize and migrate into the wound space. Cells were fixed in 4% PFA and stained with Phalloidin and pericentrin antibody followed by specific secondary antibody detection. Finally, nuclei were counterstained with DAPI and cells photographed with an inverted fluorescence microscope (See detailed antibody list and working dilutions in S1 Table).

### Chromosomal abnormalities

iPSC were expanded in feeder-free conditions using mTeSR™1 medium (Stemcell technologies) following the manufacturer’s recommendations. Upon reaching 80% confluence, iPSC colonies were fixed in 4% PFA for 5 min, washed in PBS and stained with DAPI (1μg/mL) for 5 minutes. Stained cells were imaged with a fluorescence microscope and anaphase bridge indexes (ABI) were scored as described[15]. For centrosome enumeration, cells were fixed and stained with anti-human pericentrin antibody as described above.

### Intestinal differentiation

Differentiation of control and mutant iPSC lines into intestinal tissue was performed as described[16] with the following modifications. First, iPSC lines were cultured in feeder-free conditions for at least 3 passages. iPSC colonies were then triturated into 1–2 mm fragments, transferred onto Matrigel-coated 24-well dishes, and cultured in mTeSR™1 medium for three to four days. Once cells reached 80% confluence, intestinal differentiation was begun by aspirating mTeSR™1 media and adding endoderm differentiation media containing Activin A on days 1, 2, and 3, as described [16]. Fresh mid/hindgut differentiation media was changed daily from day 3 to day 8, as indicated in the text, and consisted in RPMI-1640 media supplemented with human recombinant FGF4 (500 ng/ml) and the GSK3β inhibitor CHIR99021 (3 μM), instead of Wnt3a protein. Starting as early as two days after exposure of cells to mid/hindgut differentiation media, intestinal spheroids were collected and embedded into ice-cold intestinal Matrigel (Matrigel® Matrix. Corning: supplemented as the intestinal growth media below). Intestinal matrigel drops containing the Spheroids were placed onto 24-well plates and allowed to solidify by incubating them at 37°C for 10 min. Fresh DMEM/F12 based intestinal growth media containing Rspondin1 (500ng/mL), Noggin (100ng/mL), and EGF (100 ng/mL) was added and replaced every 4 days. iPSC-derived “intestinal organoids” (Passage 1) were split approximately every other week or used for experiments.

### Flow cytometry

At day 3 of the differentiation protocol, cells were harvested with Gentle Cell Dissociation Reagent (STEMCELL Technologies Inc.), washed with PBS containing 0.2% of Bovin Serum Albumin (BSA) and stained using conjugated antibodies against markers CXCR4 and c-KIT (S1 Table). The efficiency of definitive endoderm differentiation was measured through multicolor cytometric analysis using a FACSCalibur flow cytometer (BD Biosciences).

In the same way, flow cytometry analysis was used to quantify differences on the proliferation rates between iPSC and HIOs samples from controls and FAP patients following the recommendations of the Click-iT EdU Alexa Fluor 488 Imaging Kit (Life Technologies).

### Immunofluorescence and microscopy

At day 8 of differentiation, cultured cell layers containing three-dimensional (3D) emerging structures were fixed with 4% paraformaldehyde (PFA) in PBS during 15 minutes and washed 3X for 5 minutes with TBST buffer. Cells were permeabilized with 1% Triton X-100 for 10 minutes. After washing with TBST, cells were blocked with 4% Normal Donkey Serum (Sigma-Aldrich, D9663) during 30 minutes and stained with specific antibodies for CDX2 and SOX17. After 1hour of incubation, cells were washed with TBST, followed by incubation with appropriate antibodies conjugated to Alexa Fluor 488 and Cy3 during 45 minutes. DNA was detected with DAPI. All the steps were performed at room temperature (RT). Well images were taken with the Keyence BZ-X710 fluorescence microscope.

At day 21, 3D matrigel containing developed organoids was pipetted into a 15 ml Falcon tube and fixed with fresh 4% PFA in PBS at 4°C overnight. The next day, organoids were recovered from the diluted matrigel, washed on PBS, and stained following the steps as previously indicated for cultured cells, with specific primary antibodies against the intestinal markers CDX2 and VILLIN, and Hoechst for DNA detection. Whole organoids were mounted on cavity slides (Eisco) and imaged with a Zeiss LSM 700 confocal microscope.

After 40 days, organoids were fixed in 4% PFA in PBS at 4°C overnight. Samples were washed in cold PBS, rinsed six times in 7.5% sucrose in PBS (10 minutes each) and incubated in 30% sucrose on the rotor at 4°C overnight. Next day, samples were embedded in O.C.T and frozen with liquid nitrogen. Frozen sections were permeabilized and blocked as described before and stained with the intestinal markers CDX2, villin and lysozyme as well as antibodies against β-catenin and EdU for proliferation analysis (Antibodies and dilutions are described on S1 Table).

### RNA isolation and quantitative real-time RT-PCR

Total RNA from 2D cultured cell and 3D organoids was isolated using the RNeasy Kit (Qiagen) following the manufacturer’s recommendations. Reversed transcription of 1μg of total RNA from each sample was carried out with the SuperScript™ III First-Strand Synthesis System (Invitrogen). The resulting cDNA was diluted to a final concentration of 50 ng/μl and gene expression was detected with specific taqMan Probes (Thermo Fisher Scientific) (S2 Table) with the StepOnePlus R-T PCR system (Applied Biosystems). The delta-delta Ct method was used to quantify the fold change expression related to undifferentiated iPSC using GAPDH as the housekeeping gene for sample normalization.

### Digital genome sequencing (DGE)

Gene expression data was generated via a 3' DGE protocol for high-throughput single cell RNA barcoding and sequencing[17], run and aligned (GRCh37/hg19 assembly) at the Broad Institute in Cambridge, MA. Normalization and analysis of expression data was performed using the R statistical software environment (R v.3.1.2; https://cran.r-project.org/) and Bioconductor (https://www.bioconductor.org/). Pairwise differential expression analysis between samples was performed using the edgeR package (https://bioconductor.org/packages/release/bioc/html/edgeR.html). Multiplicity correction was performed by applying the Benjamini-Hochberg method on p-values to control the false discovery rate (FDR), with top genes sorted by FDR and called as significantly differentially expressed when meeting thresholds of p value<0.05. Heatmaps were generated using gplots(http://www.inside-r.org/packages/cran/gplots/docs/heatmap.2) and RColorBrewer(https://cran.r-project.org/web/packages/RColorBrewer/index.html). Expression data of significant genes was log2 adjusted and samples were clustered via Pearson correlation and average linkage. All raw and processed data has been deposited in GEO (GSE99821).

### Ingenuity® pathway analysis (IPA®)

IPA®, QIAGEN Redwood City, www.qiagen.com/ingenuity) software (build version: 389077M, content version: 27821452) was used to generate functional networks and analyze signal transduction pathways. The functional networks and signal transduction pathways of IPA are predicted on the basis of known gene–gene (protein–protein) and functional interactions based on information contained in the Ingenuity Pathway Knowledge Base.

### Statistical methods

Student's t-tests were performed for statistical analysis. Statistically significant differences from the wild-type control are indicated by asterisks: *p< 0.05; **P < 0.01.

## Results

### A mini-library of FAP-specific iPSC

**Fig 1:**
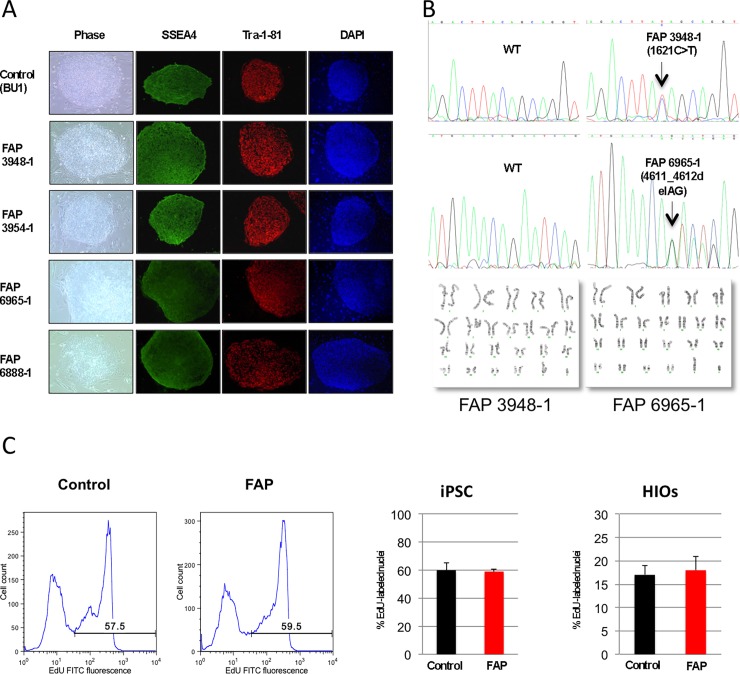
Characterization of wild-type and FAP iPSC. (A) iPSC were generated with the constitutive EF1a-STEMCCA vector expressing the four reprogramming factors Oct4, Klf4, Sox2 and c-Myc and containing a loxP site within the 3' LTR that allows excision of the entire cassette following exposure to Cre recombinase. Transgene-free iPSC clones generated with STEMCCA display the typical human ESC morphology and stain positive for pluripotency markers. (B) Sequence analysis of the *APC* gene in iPSC clones BU1 (WT), FAP 3948–1 and FAP 6965–1. Arrows indicate a C>T change resulting in the conversion of glutamine 541 of APC to a stop codon (FAP 3948–1), and a heterozygous 2 bp deletion in codon 1537 that results in a frameshift mutation that leads to a premature termination at proline 1542 (FAP 6965–1). G-banding chromosome analysis reveals normal diploid number of chromosomes (2n = 46) in two representative FAP-iPSC lines. (C) iPSC and intestinal organoids (HIOs) were pulsed with EdU, and then fixed, permeabilized, and stained with the Click-iT reaction. Percentages of cells in S + G2/M phase are indicated in the graphs. Bar graph shows average +/- SD of two independent lines done in triplicate.

Fibroblasts from four previously diagnosed FAP individuals were obtained from Coriell and reprogrammed using the STEMCCA system to generate three independent iPSC lines, as described [12, 18] (Fig 1A). Two of the patient lines (FAP 3948–1 and FAP 3954–1) contain a C>T mutation in the coding sequence of the *APC* gene at position 1621 and the other two (FAP 6965–1 and FAP 6888–1) contain a AG deletion at position 4611 (Fig 1B), both inducing production of truncated versions of APC protein. Overall, there were no major phenotypic differences between the FAP-iPSC compared to normal cells in terms of proliferation rate. We did observe an unusual growth pattern of the FAP-iPSC clones (“ring” morphology, S1 Fig) when the cells were expanded on feeders, likely a consequence of spontaneous differentiation. This morphology disappeared when the cells were passaged on Matrigel under feeder-free conditions, however and as detailed below, the mutant cells showed increased spontaneous differentiation. Clones FAP 3948–1 and FAP 6965–1 showed the more robust growing conditions (easier to passage, less spontaneous differentiation, healthier morphology) and we focused on these two clones for our experiments. In an attempt to see if mutations in APC affected the proliferation rate of the cells we quantified EdU uptake and incorporation into newly synthesized DNA. As shown in Fig 1C, there was no difference between normal and FAP-specific clones.

**Fig 2:**
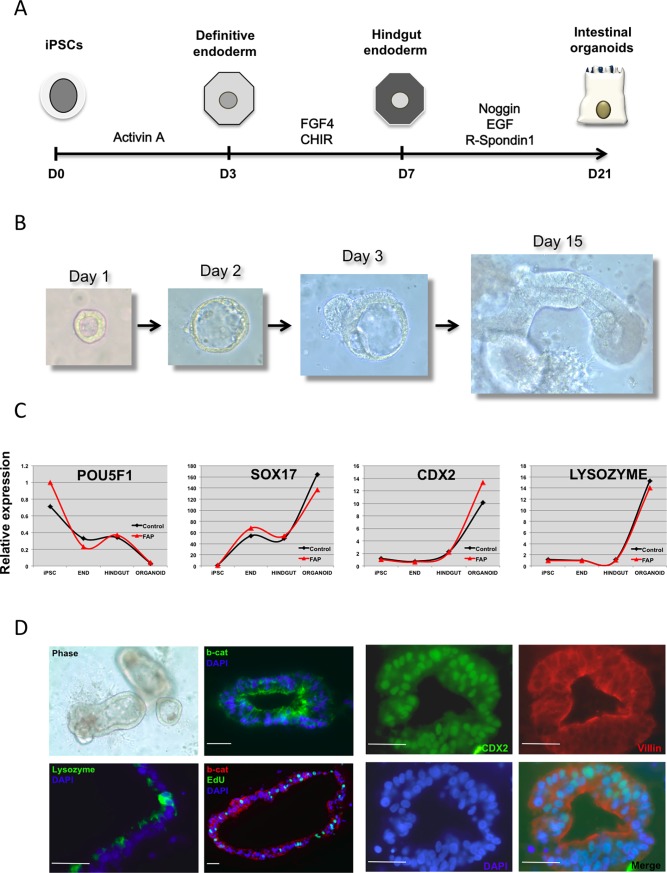
Directed differentiation of iPSC into intestinal organoids. (A) Schematic of the protocol employed to induce intestinal differentiation from iPSC. Cells are first induced to form endoderm followed by hindgut specification and formation of intestinal organoids in matrigel. (B) Bright field images showing formation of a representative iPSC-derived intestinal organoid upon incubation in matrigel. (C) Real-time RT-PCR analyses of differentiating cells at different stages demonstrate robust endoderm induction and hindgut specification followed by efficient generation of intestinal organoids from both normal control and FAP-specific iPSC lines. (D) Representative micrographs and immunofluorescence images of the intestinal organoids derived from an FAP-iPSC reveal formation of 3D epithelial structures with clear expression of intestinal markers CDX2, VILLIN and LYSOZYME. Edu staining allows visualization of proliferating cells within the organoids. β-CATENIN staining shows mostly membrane and cytoplasmic pattern.

If *APC* in heterozygosity is sufficient to induce changes in β-catenin signaling, FAP-specific iPSC should display a spontaneous increase in expression of *WNT* target genes as well as spontaneous differentiation toward mesendodermal lineages [19]. However, when comparing normal vs FAP cells we found gene expression of several *WNT* target genes (including *FOXA2*, *AFP*, *MSX1*, *GATA4* and *BRACHYURY*) to be variable between iPSC lines from different donors. This inter-individual variability was confirmed by comparing iPSC lines derived from three healthy donors (S2 Fig). We speculated that differences mediated by *APC* mutations may become more evident upon differentiation into intestinal epithelium, as APC expression increases upon gut differentiation (S3 Fig). For this purpose we utilized a protocol developed by Wells and colleagues that employs sequential stimulation of NODAL and FGF4R /WNT3A to mimic early development and drive endodermal specification first into mid- and hindgut prior to differentiation into intestinal epithelium (Fig 2A) [20]. Indeed, when exposed to these conditions we were able to obtain definitive endoderm and intestinal specification, with formation of 3D human intestinal organoids (HIOs) upon expansion in matrigel embedded cultures (Fig 2B) from both normal and mutated *APC* iPSC. Expression of *SOX17*, *CDX2* and *LYSOZYME* followed the expected developmental pattern (Fig 2C) with upregulation of *SOX17* in endodermal cells followed by upregulation of the intestinal markers. Similar to our findings in undifferentiated iPSC, comparison between the normal and the *APC*-mutant iPSC-derived HIOs did not reveal statistically significant differences in proliferation measured by EdU incorporation (Fig 1C) or expression of WNT target genes (S3 Fig). However, we consistently observed that FAP-specific iPSC evidenced faster differentiation towards endoderm than control lines, with expression of SOX17 already detectable after short (3.0 days) NODAL activation (Fig 2C and S4 Fig). This finding appears to correlate with increased expression of *CDX2*, more evident in the later stages of intestinal specification (Fig 2C). Immunohistochemistry confirmed efficient expression of CDX2 (95% of cells were CDX2+), VILLIN and β-CATENIN in intestinal organoids (Fig 2D).

In order to minimize the potential noise created by inter-individual genetic background variability, we decided to create a gene editing-based *APC*-specific iPSC platform that would allow the comparison of isogenic *APC* mutant vs normal iPSC.

### Isogenic platform of normal and *APC*-mutant iPSC

**Fig 3:**
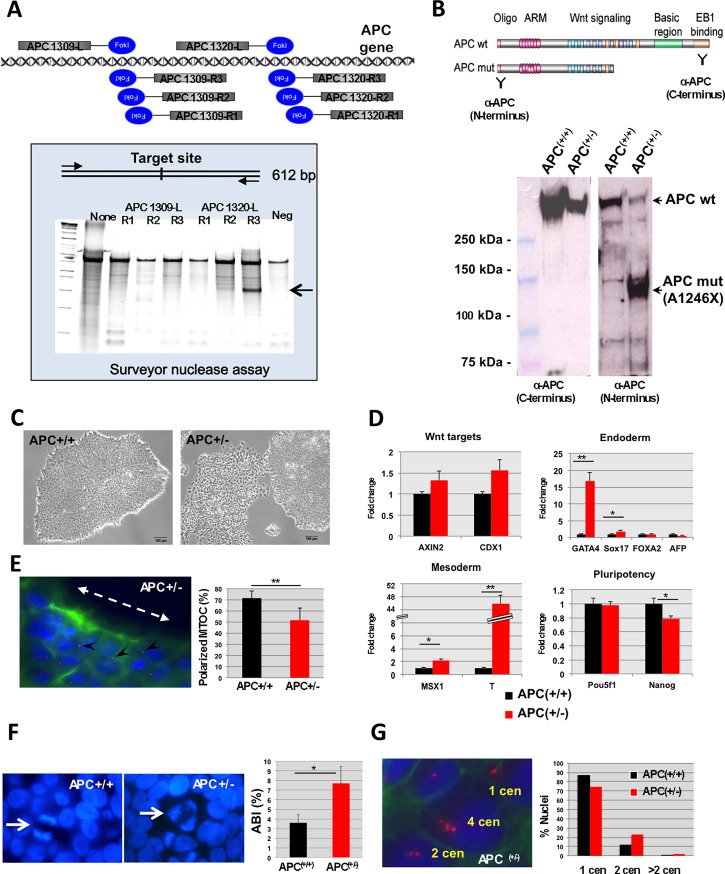
Generation of isogenic *APC*+/- iPSC line and its effect on cell polarity and chromosomal instability. (A) We designed several pairs of site-specific TALENs targeting the region surrounding codon 1309 of *APC*, a known mutational hotspot. The efficiency of gene modification by NHEJ was evaluated by the Surveyor Nuclease Assay. (B) Schematic of the two APC proteins expressed by the heterozygous mutant iPSC line based on DNA sequencing and binding recognition of the antibodies used for Western blot. Western blot confirms expression of both the WT and truncated versions of APC. (C) Bright field images comparing the *APC*+/+ vs the *APC*+/- human iPSC colonies showing spontaneous differentiation of the mutant cells. (D) Mutant iPSC show transcriptional changes reminiscent of increased endogenous *WNT* signaling, as determined by TaqMan qPCR analysis of endoderm, mesoderm and *WNT* target genes.*p<0.05, **p<0.01. (E) A scratch-induced cell migration assay reveals impaired centrosome reorientation in mutant iPSC-derived fibroblasts compared with the control, with a significant reduction in polarized MTOC. (F-G) Also consistent with the role of APC in microtubule dynamics, *APC* mutant iPSC display an increase in chromosomal instability, as evidenced by increase in abnormal anaphases (quantitated by Anaphase Bridge Index or ABI) and higher number of centrosomes (cen).

In order to generate an isogenic system we designed TALENs targeting position 1320 of the *APC* coding sequence (Fig 3A), a known hotspot commonly mutated in FAP patients [2]. We chose BU1 iPSC as our targeting clone, which has been extensively characterized and used in our lab at the CReM [21]. After four independent attempts and despite consistently observing an efficiency of ~15–20% of TALEN-induced indels, the overall efficiency of a not-in-frame heterozygous mutation was significantly low (1 out of 16), strongly suggesting that having deleterious mutations in *APC* induces a selection disadvantage to the survival, reprogramming, or outgrowth of iPSC in culture. Nevertheless, we were able to obtain and expand a karyotypically normal iPSC isogenic clone containing a 140 bp deletion that introduced a premature stop codon at position 1246 with translation of a truncated APC protein lacking part of the WNT signaling domain and the entire Basic Region and EB1 binding domain in the C-terminus end (Fig 3B). The *APC* +/+ and *APC*+/- iPSC isogenic lines were then expanded and maintained in identical culture conditions for the rest of the studies. The first obvious cellular effect of *APC* dysregulation was the spontaneous differentiation phenotype of the *APC* +/- clone in culture (Fig 3C), which was confirmed molecularly by upregulation of several primitive streak, mesodermal and endodermal genes (Fig 3D) compared to the *APC* +/+ line. This was accompanied by an increase in *WNT* target gene expression, including *AXIN2* and *CDX1* (Fig 3D).

### *APC* haploinsufficiency affects the phenotype of isogenic iPSC and their differentiated progeny

The known functions of APC in cellular motility and cytoskeletal integrity [22, 23] prompted us to investigate whether expression of a defective copy of *APC* could affect cell polarity. For this purpose we adapted a protocol for the differentiation of iPSC into fibroblasts (as demonstrated by positive CD90/Thy-1 staining, not shown) and performed a classic “wound repair assay”. First, iPSC-derived fibroblasts were grown to confluence and then a mechanical “scratch” was induced with a pipette tip. Fibroblasts then tend to grow towards the “wound” by polarizing the cells and then migrating to cover the defect. Staining the cells with phalloidin, pericentrin and DAPI enables the quantification of the location of the microtubule-organizing center (MTOC) relative to the nucleus as a measurement of cell polarity. As shown in Fig 3E, *APC* mutant cells showed a significant decrease in the percentage of polarized MTOC. As APC has also been implicated in the regulation of chromosomal segregation and the formation of the mitotic spindle, with *APC* mutations causing chromosomal abnormalities [24], we tested two independent assays to detect chromosomal aberrations. Indeed, *APC* mutant cells showed a significant increase in abnormal anaphases (measured as Anaphase Bridge Index or ABI [15]) with almost double the number of abnormal anaphases (Fig 3F). Similarly, while most normal *APC*+/+ cells contained 1 centrosome and rarely 2, *APC* mutant cells showed a significant increase in cells containing 2 centrosomes and even 4 centrosomes, which was never observed in the normal cells (Fig 3G).

**Fig 4:**
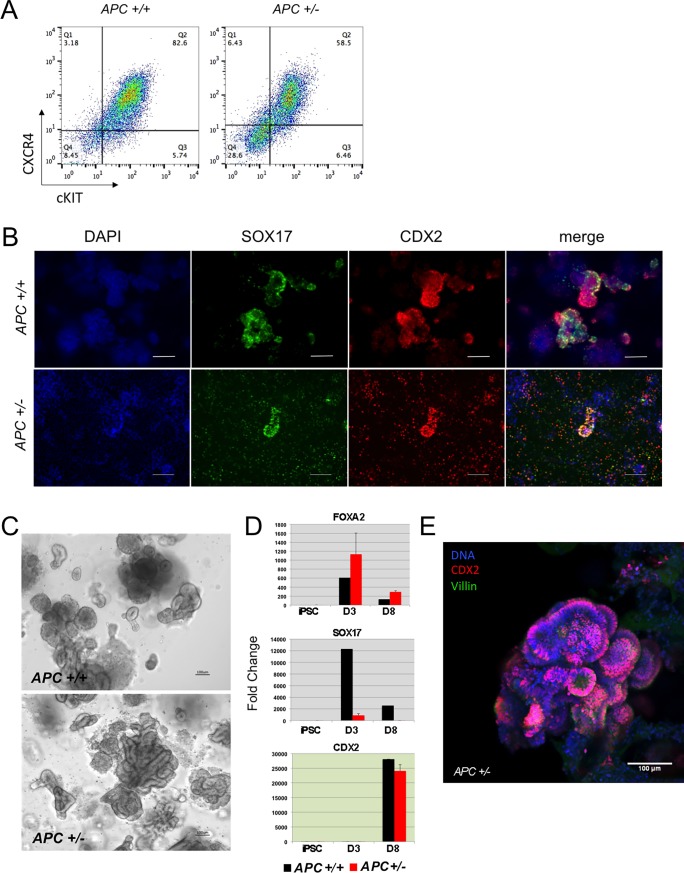
Intestinal differentiation of *APC*+/+ vs *APC*+/- mutant iPSC. (A) Efficient endoderm specification by the *APC* +/+ and *APC* +/- iPSC isogenic lines as evidenced by cKit and CXCR4 expression. (B) Immunohistochemistry of cells at day 8 of differentiation shows similar patterns of SOX17 and CDX2 expression. (C) Bright field images show representative intestinal organoids upon incubation in matrigel. (D) Real time RT-PCR shows similar dynamics of differentiation with upregulation of *FOXA2* and *SOX17* during endodermal specification (Day 3: D3) and robust upregulation of *CDX2* in hindgut (Day 8: D8). (E) Whole mount immunohistochemistry of a representative organoid from the *APC* +/- iPSC line showing CDX2 (in red) and VILLIN (in green) expression counterstained with DAPI (blue).

Next, we induced the differentiation of the *APC*+/+ and *APC*+/- into intestinal organoids, following the protocol described above. As shown in Fig 4, we were able to obtain efficient differentiation with the *APC* mutant line, first into endoderm (Fig 4A) with significant *CDX2* expression upon addition of FGF4 and WNT3A as observed with the WT isogenic line (Fig 4B) and robust formation of intestinal organoids upon culture in matrigel (Fig 4C–4E). Furthermore, and in concordance with what we observed with the FAP lines, staining the organoids on day 30 post differentiation with EdU/DAPI revealed similar proliferation rates in mutant and WT cells (not shown).

### *APC* haploinsuffciency is associated with a definable gene expression profile, particularly associated with altered metabolic reprogramming and dysregulation of lipid metabolism and cadherins

**Fig 5:**
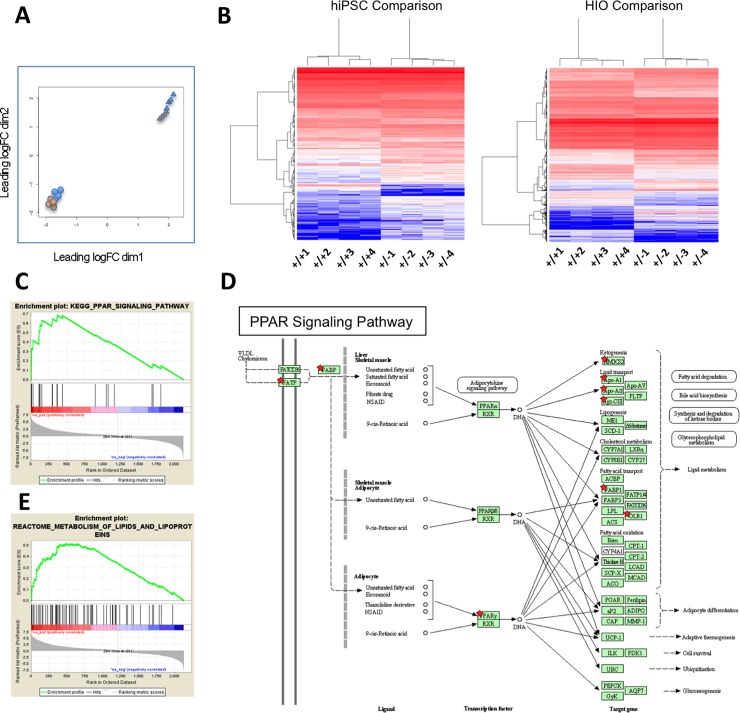
DGE analysis of *APC*+/+ vs *APC*+/- before and after differentiation into intestinal organoids. (A) Multidimensional Scaling plot (MDS) shows whole genome transcriptome analysis of four independent *APC*+/+ (orange) vs four independent *APC*+/- (blue) iPSC subclones before (circles) and after differentiation (triangles) into intestinal organoids. (B) Unbiased hierarchical clustering heatmaps of undifferentiated (hiPSC) or differentiated intestinal organoids (HIO) using pairwise differential expression analysis between *APC*+/+ vs *APC*+/- samples, with an FDR cutoff of at least 0.05. (C) GSEA of KEGG pathways showing the top most affected pathway based on the differentially expressed genes. (D) Schematic of the PPAR Signaling pathway. Red stars denote individual components of the pathway that appear differentially expressed between *APC*+/+ vs *APC*+/-. (E) GSEA of metabolic pathways showing the lipid metabolism pathway is affected in *APC* mutant cells.

In order to study whether a truncated form of APC is associated with changes in the gene expression landscape of both undifferentiated iPSC and their intestinal progeny, we sought to perform RNA seq by digital gene expression (DGE). Similarly to SAGE, DGE allows sequencing of most RNA transcripts [17], including low abundant ones that can be missed when using hybridization-based Affymetrix chip arrays. Not surprisingly, the first analysis done using Multidimensional Scaling plot (MDS) showed a clear separation between the undifferentiated cells and the HIOs (Fig 5A). More importantly, this analysis demonstrated that the presence of one *APC* mutated allele changed the gene expression signature of the cells, separating *APC* WT from *APC* mutant cells, and that the effect of the mutation appeared to be more pronounced in the intestinal organoids than in undifferentiated iPSC (Fig 5A). Indeed, pairwise differential expression analysis between normal vs mutant samples at each time point (using the edgeR package with an FDR cutoff of at least 0.05) showed 2124 differentially expressed genes between normal and mutant HIOs, compared to only 322 genes differentially expressed between normal and mutant undifferentiated cells (S3 Table). Furthermore, these expression differences resulted in separate clustering of the normal vs wildtype cells at each time point when analyzed by unsupervised hierarchical clustering (Fig 5B).

Upon analysis of each set of differentially expressed genes for potential differences in epithelial or signaling programs, we observed that Cadherin 17 (*CDH17*), was the number 13 most highly dysregulated among the 2124 misexpressed genes in HIOs (with an FDR value of 1.54E-81). Cadherin 17, also known as IL Cadherin or Intestinal-Liver Cadherin is a member of the cadherin superfamily that appears to be expressed primarily in the intestinal tract and its protein may play a role in the morphological organization of the intestine and liver. Remarkably, abnormal CDH17 expression has been described as a diagnostic marker of adenocarcinomas of the digestive system [25], with higher sensitivity than CDX2 [26]. Another gene that showed a striking increase in *APC* mutant HIO was Isocitrate dehydrogenase 1 (*IDH1*) (logCPM 9.77956, p-value 4.42E-56 and FDR 2.94E-53). One of the top 5 canonical pathways that was found by Gen Set Enrichment Analysis (See below) within the HIO gene set was oxidative phosphorylation (p-value = 5.74E -11, S1 Table). In that regard, another gene of interest found to be dysregulated was Lactate dehydrogenase A (LDHA) (logCPM 9.20493, p-value 6.05E-13 and FDR 5.20E-11). LDHA is considered as critical for cancer cells since it catalyzes the conversion of pyruvate to lactate. It is elevated in several cancers and has high potential as a diagnostic, predictive as well as therapeutic target for new anticancer treatments [27–29]. Upregulation of *IDH1* and *LDHA* in *APC* mutant HIO might indicate an early metabolic reprogramming event associated with *APC* mutations that has not been studied yet. Finally, *VIMENTIN*, which also appeared dysregulated in the *APC* mut HIO (logCPM 9.32429, p-value 1.59E-30 and FDR 4.42E-28) is overexpressed in several cancers including gastrointestinal cancers and correlates with poor prognosis and accelerated growth and invasion of cancer cells. In addition, *VIMENTIN* is a marker for epithelial-mesenchymal transition (EMT) and is currently being evaluated as a potential target for anti-cancer therapy [30, 31].

The top genes found upregulated in the DGE analyses of the isogenic *APC* heterozygous iPSC line were confirmed to be overexpressed also in HIOs from FAP patient-specific iPSC (S5 Fig). Real-time RT-PCR of representative genes reinforced the evidence pointing towards a fatty acids altered metabolism, being *FABP1*, *ALDH1A1* and *APO1* overexpressed more than 100 fold (S5 Fig).

### GO, GSEA and Ingenuity pathway analysis (IPA) of HIO gene sets

Analysis of the differentially expressed genes was done using three independent platforms, Gene Ontology (GO), Gene Set Enrichment Analysis (GSEA) and Ingenuity Pathway Analysis (IPA) and all showed similar results. Among the dysregulated KEGG pathways associated with *APC* mutations, the PPAR signaling pathway appears at the top of the list (Fig 5C). Indeed, within this pathway *APC* appears to modulate several genes that participate in adipocyte function and lipid metabolism, including *FATP*, *FABP*, *PPARγ*, *FABP1*, *OLR1* and several lipid transporters of the APO family (Fig 5D). Validating these findings, GSEA analysis revealed the *APC* mutation was associated with abnormal lipid function (Fig 5E).

**Fig 6:**
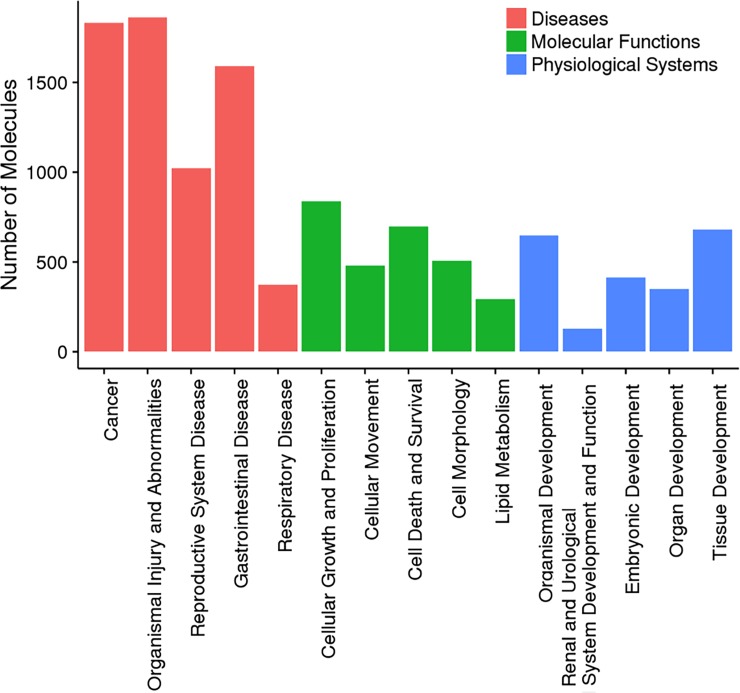
Ingenuity pathway analysis (IPA) of DGE data. Whole genome expression data was analyzed using IPA focusing on Diseases and Disorders (Red), Molecular and Cellular Functions (Green) and Physiological System Development Function (Blue). Number of genes (Number of Molecules) within the top five categories are shown in graphs.

In order to further validate the pathways affected in *APC*+/- HIO, Ingenuity® Pathway Analysis (IPA) was used. A total of 2124 differentially expressed genes were uploaded to IPA for core analysis. Out of those, 2113 genes IDs were successfully mapped. As shown in Fig 6, we analyzed the top 5 diseases and bio functions and scored them as a percentage of the Top 5 hits. We observed cancer (p-value 2.61E-05–9.10E-33) and gastrointestinal diseases (p-value 2.43E-05–5.11E-17) [within Top Disease and Disorder subset], cellular growth and proliferation (p-value 3.13E-05–1.35E-27), cellular movement (p-value 3.19E-05–3.46E-23), cellular survival (p-value 2.53E-05–9.24E-23) and lipid metabolism (p-value 2.76E-05–4.22E-14) [within Top Molecular and Cellular Functions subset] and organismal development (p-value 3.12E-05–4.57E-21), tissue-, organ- and embryonic-development (all with similar p-value of 2.74E-05–1.35E-19) [within Top Physiological System Development and Function subset] were the top hits within the Top Disease and Bio Functions categories of APC+/- HIO-associated genes. For each of the top 5 Disease and Bio Function, we also estimated the percentage of genes that were involved in the process as compared to the total number of total genes used in the core analysis dataset (Table 1). We observed that cancer (86.2%), cellular growth and proliferation (39.4%) and organismal development (30.4%) had the highest number of common genes within each subset of Top Disease and Bio Functions.

**Table 1:** Summary of ingenuity pathway analysis.

Name	# Molecules	# Molecules % of total 2125 genes	p-value
**Top 5 Diseases and Disorders**			
Cancer	1832	86.21176	2.61E-05–9.10E-33
Organismal Injury and Abnormalities	1861	87.57647	3.19E-05–9.10E-33
Reproductive System Disease	1023	48.14118	2.61E-05–2.36E-23
Gastrointestinal Disease	1590	74.82353	2.43E-05–5.11E-17
Respiratory Disease	372	17.50588	3.19E-05–9.00E-14
**Top 5 Molecular and Cellular Functions**			
Cellular Growth and Proliferation	839	39.48235	3.13E-05–1.35E-27
Cellular Movement	481	22.63529	3.19E-05–3.46E-23
Cell Death and Survival	696	32.75294	2.53E-05–9.24E-23
Cell Morphology	506	23.81176	2.53E-05–1.66E-16
Lipid Metabolism	292	13.74118	2.76E-05–4.22E-14
**Top 5 Physiological System Development and Function**			
Organismal Development	647	30.44706	3.12E-05–4.57E-21
Renal and Urological System Development and Function	130	6.117647	2.33E-05–4.57E-21
Embryonic Development	412	19.38824	2.74E-05–1.35E-19
Organ Development	348	16.37647	2.74E-05–1.35E-19
Tissue Development	682	32.09412	2.74E-05–1.35E-19

## Discussion

The recent establishment of in vitro 3D culture approaches to model intestinal morphogenesis, either from intestinal crypts [32] or from pluripotent stem cells [20] had opened new exciting opportunities to study in vitro intestinal tumorigenesis. Recent studies using in vitro organoids grown from different intestinal tumors [33, 34] showed a strong correlation, functionally and molecularly with their primary tissue counterparts, providing a strong argument in favor of the potential for in vitro systems to model intestinal disease. In the case of iPSC, access to intestinal epithelial cells offers a unique approach to study in a cell intrinsic manner the earliest cellular and molecular events associated with APC mutagenesis and its potential role in CRC development. Taking advantage of the fact that patient somatic cells are *APC* heterozygous, we sought to recapitulate the primordial developmental state of gut epithelia and study the cellular and molecular “milieu” that may provide a bona fide environment for the development of intestinal tumors. We demonstrated that FAP patient iPSC were able to differentiate into gut organoids, although many differences found in the intestinal-like tissues developed from normal or heterozygous *APC* iPSC were certainly blurred by the inter-patient variability. Once we moved into the isogenic system, we were able to find robust and significant differences between normal and *APC* mutant intestinal epithelial cells. It is relevant to emphasize that studies with the FAP lines were also limited by the unstable nature of the *APC* mutant lines. Spontaneous differentiation was always evident in our cultures requiring extra care in the maintenance and passaging of the lines, and was further supported by the faster induction of markers such as *SOX17* and *CDX2* in the APC mutant cells.

Another interesting finding relates to the gene editing approach to create the *APC* mutant cells. As mentioned above, even though we had a relatively high efficiency of TALEN induced DNA double strand breaks (~15–25%), the overall efficiency of obtaining non-conserved mutations bearing truncated versions of APC was very low (~6%). As a matter of fact, after screening more than 400 colonies we were never able to recover a line with homozygous mutations in both *APC* alleles, strongly suggesting that APC is a key factor in the maintenance of iPSC lines in their pluripotent state. Our findings that *APC* in heterozygosity was enough to induce spontaneous differentiation of the iPSC could indicate that even if our TALENs were able to create homozygous *APC* mutant lines, they were lost to spontaneous differentiation before forming colonies. This is supported by the existence of a limited number of reports describing *APC* mutant lines, which were all done on mouse ESC expressing either hypomorphic mutant variants of APC [35] or specifically overexpressing β-catenin *APC* mutations [36]. These studies showed that abnormal catenin regulation induced dysregulated in vitro differentiation (and not proliferation), findings in keeping with our *APC* heterozygous human iPSC line. Interestingly, recent studies describing a novel differentiation protocol to derive colonic organoids from human iPSC found that FAP-specific iPSC had a significant increase in proliferation as measured by percentage of ki67 positive cells [37]. Whether this difference is due to their specific protocol or to other differences in culture conditions is unclear.

The finding that *APC* in heterozygosity has such an impact on the phenotypic and molecular characteristics of our iPSC and their intestinal progeny is not completely unexpected. Several studies have previously shown that changes in *APC* dosage can affect cellular motility, migration and polarity [24, 38, 39], and can induce the development of early intestinal neoplasms [40]. Our data strongly support the role of APC in cell motility and polarity as evidenced by the migratory defect evidenced in the APC mutant fibroblasts. In addition, the presence of chromosomal aberrations (evidenced both by the higher number of abnormal anaphases as well as centrosomes) emphasizes a novel mechanism by which changes in APC function can initiate chromosomal instability and tumorigenesis.

There is a renewed interest in the area of cancer metabolism with metabolic reprogramming coined as a hallmark of cancer based on the discovery of mutations and alterations in *IDH1* and *IDH2* along with succinate dehydrogenase (*SDH*), fumarate hydratase (*FH*), and pyruvate kinase M2 (*PKM2*) [41, 42]. *IDH1/2* mutations have been observed in several solid and blood cancers including colon cancer. The metabolic activity of IDH1/2 has been tied to cancer epigenetic mechanisms. Several ongoing studies have started targeting these *IDH1/2* metabolic genes as selective targets for new anticancer therapeutics [43]. Metabolic switch from oxidative phosphorylation to increased glycolysis even under hypoxic conditions (termed Warburg effect) is a central biochemical feature of cancer cells.

### Conclusions

Our studies show that a truncated form of APC induces a significant effect on the cellular and molecular features of iPSC and their differentiated progeny. This effect is exacerbated upon differentiation into intestinal organoids with a major impact on the gene expression profile of the cells, and the emergence of a molecular signature resembling several features of intestinal tumorigenesis and abnormal metabolism. Further studies using cells with homozygous mutations mimicking the correspondent loss of heterozygosity (LOH) present in advanced intestinal tumors is warranted.

## Supporting information

S1 Fig Isogenic WT and APC +/- iPSC colonies. Representative micrographs showing bright field (top rows) and Alkaline Phosphatase staining (bottom rows) evidence abnormal colony growth pattern in APC+/- colonies compared to the WT isogenic line (BU1).
(PDF)

S2 Fig Spontaneous differentiation in human iPSC. Expression of endodermal genes measured by qRT-PCR comparing WT vs FAP iPSC lines (top graph) or three independent control lines (bottom graph).
(PDF)

S3 Fig Expression of APC and Wnt target genes. (A) Expression of APC measured by qRT-PCR in WT (black) vs FAP (red) iPSC before (d0) and after differentiation into intestinal organoids (d40). (B) Expression of Wnt target genes in intestinal organoids. Data are mean ± SE from two independent WT and FAP lines.
(PDF)

S4 Fig FAP iPSC differentiate into HIOs faster than WT cells. Expression of the endodermal marker Sox17 overtime during differentiation of control (black) vs FAP (red) iPSC into intestinal organoids. DE: definitive endoderm. Data are mean ± SE of two independent lines.
(PDF)

S5 Fig Validation of DGE expression. qRT-PCR data of selected genes in WT vs FAP iPSC upon intestinal differentiation. Data are mean ± SE of two independent lines.
(PDF)

S1 Table List of antibodies used throughout these studies (See Materials and methods). (DOCX)

S2 Table List of TaqMan probes used in the real-time PCR experiments (See Materials and methods). (DOCX)

S3 Table Complete list of genes that are dysregulated in APC+/+ vs APC +/- iPSC before (blue) and after (red) differentiation into HIOs. Top genes sorted by FDR and called as significantly differentially expressed when meeting thresholds of p value<0.05.
(XLSX)
