# Supplementary figures and images for: Modeling APC mutagenesis and familial adenomatous polyposis using human iPS cells
Source: PLoS One. 2018 Jul 19;13(7):e0200657. doi: 10.1371/journal.pone.0200657 (PMC6053155; doi:10.1371/journal.pone.0200657)

**BU1**

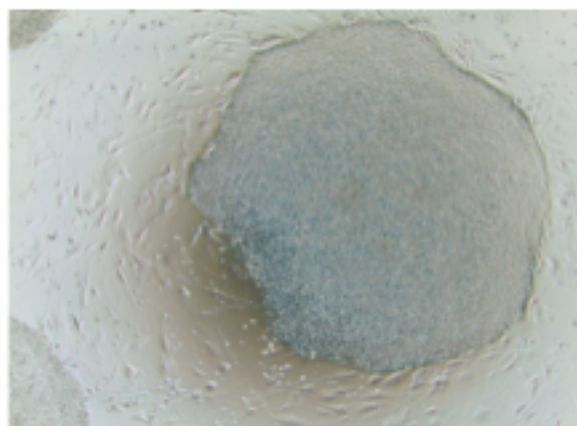

**APC+/-**

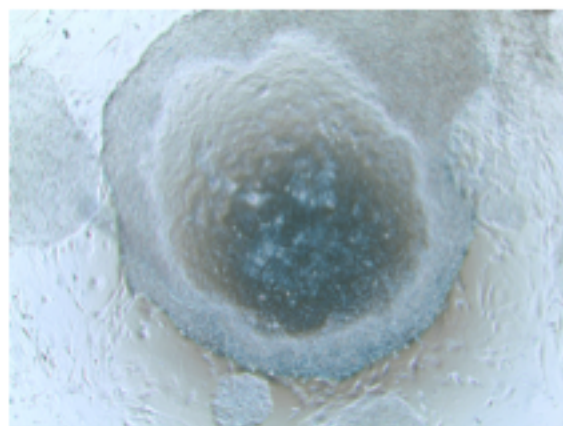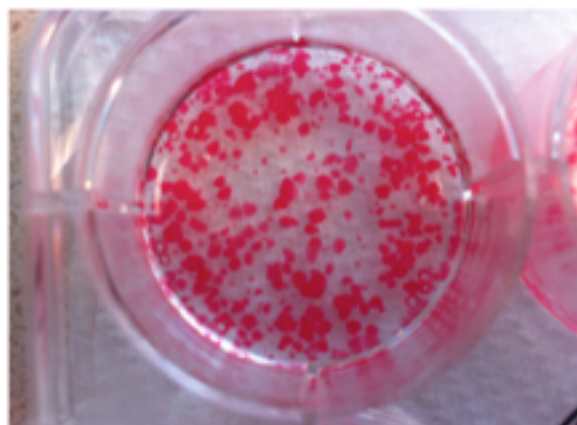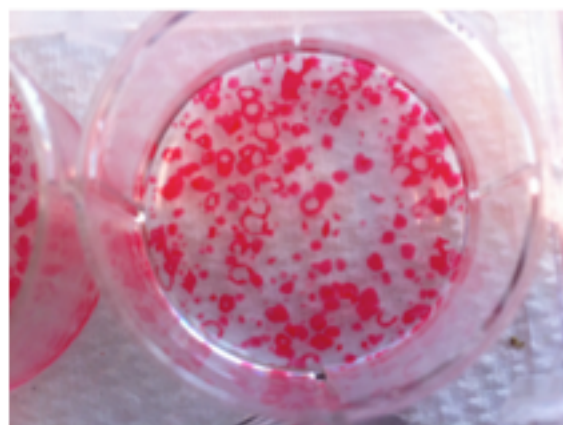

Supplement: S1 Fig — Representative micrographs showing bright field (top rows) and Alkaline Phosphatase staining (bottom rows) evidence abnormal colony growth pattern in APC+/- colonies compared to the WT isogenic line (BU1). (PDF) [file pone.0200657.s001.pdf]

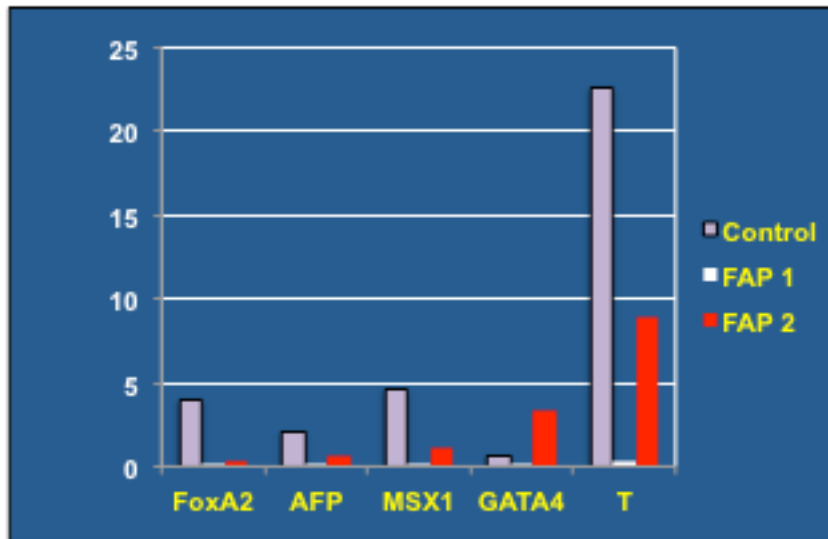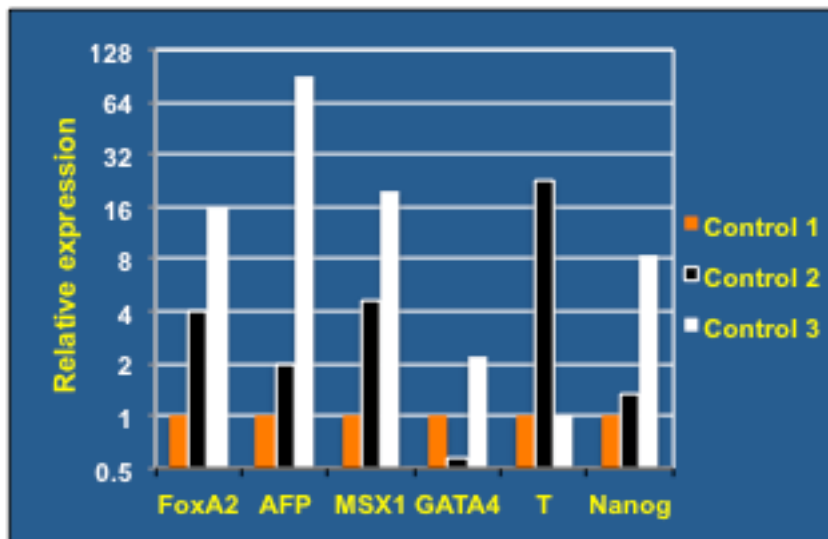

Supplement: S2 Fig — Expression of endodermal genes measured by qRT-PCR comparing WT vs FAP iPSC lines (top graph) or three independent control lines (bottom graph). (PDF) [file pone.0200657.s002.pdf]

**A**

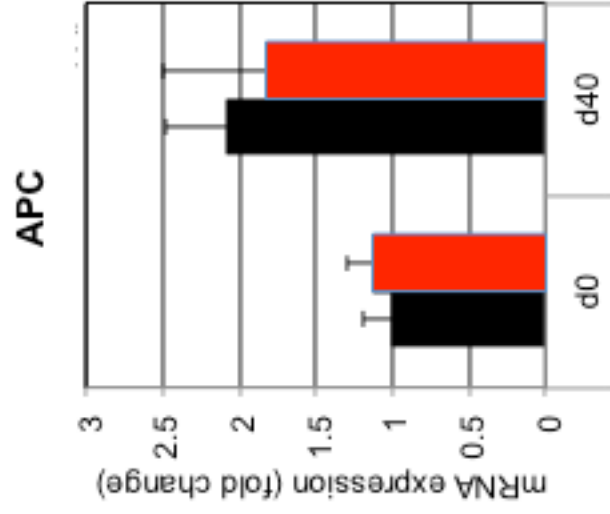

**B**

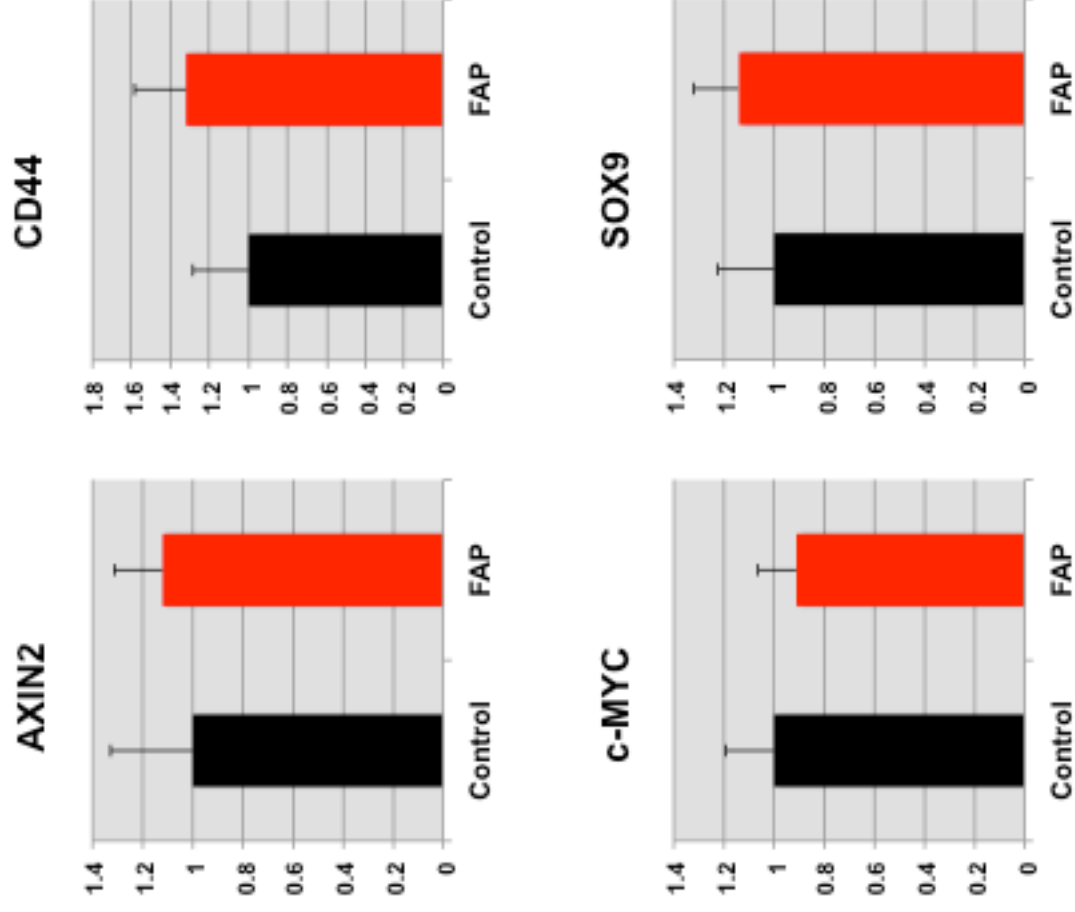

Supplement: S3 Fig — (A) Expression of APC measured by qRT-PCR in WT (black) vs FAP (red) iPSC before (d0) and after differentiation into intestinal organoids (d40). (B) Expression of Wnt target genes in intestinal organoids. Data are mean ± SE from two independent WT and FAP lines. (PDF) [file pone.0200657.s003.pdf]

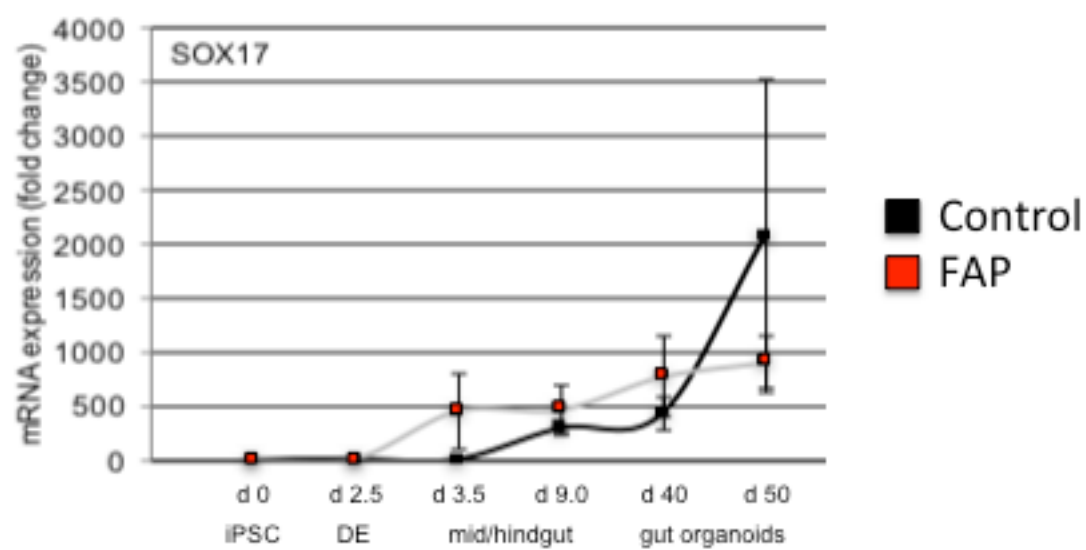

Supplement: S4 Fig — Expression of the endodermal marker Sox17 overtime during differentiation of control (black) vs FAP (red) iPSC into intestinal organoids. DE: definitive endoderm. Data are mean ± SE of two independent lines. (PDF) [file pone.0200657.s004.pdf]

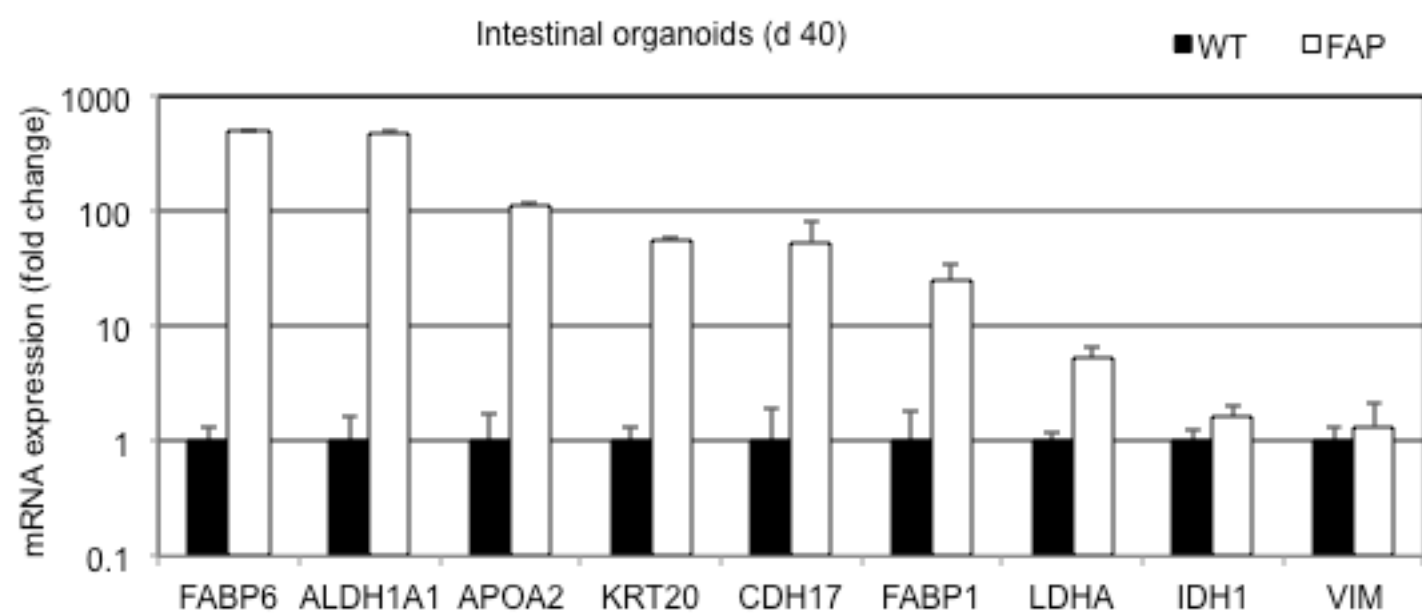

Supplement: S5 Fig — qRT-PCR data of selected genes in WT vs FAP iPSC upon intestinal differentiation. Data are mean ± SE of two independent lines. (PDF) [file pone.0200657.s005.pdf]
