# Supplementary material for: Modeling APC mutagenesis and familial adenomatous polyposis using human iPS cells
Source: PLoS One. 2018 Jul 19;13(7):e0200657. doi: 10.1371/journal.pone.0200657 (PMC6053155; doi:10.1371/journal.pone.0200657)
Supplement: S1 Table — (DOCX) [file pone.0200657.s006.docx]

**Supplementary table 1. List of primary and secondary antibodies**

| **Primary Antibodies** | **Source (cat. Number))** | **Dilution** |
| --- | --- | --- |
| Goat anti-SOX17 | R&D systems (AF1924) | 1:300 |
| Mouse anti-gamma tubulin | Abcam (ab11316) | 1:100 |
| Mouse anti-Villin | Chemicon International Inc. (MAB1671) | 1:100 |
| Rabbit anti-Beta-catenin | Abcam (ab6302) | 1:2000 |
| Rabbit anti-CDX2 | Thermofisher Scientific (MA5-14494) | 1:100 |
| Rabbit anti-Lysozyme | Thermofisher scientific (PA1-29680) | 1:50 |
| Rabbit anti-pericentrin | Abcam (ab84542) | 1:100 |
| **Secondary antibodies/dyes** |  |  |
| Alexa Fluor 488 Phalloidin | Thermofisher scientific (A12379) | 1:500 |
| DAPI | Molecular probes (P36935) | 1:2000 |
| Goat anti-rabbit Alexa Fluor 594 | Abcam (ab150080) | 1:500 |
| Donkey anti-goat Alexa Fluor 488 | Thermofisher scientific (A-11055) | 1:500 |
| Donkey anti-mouse DyLight 488 | Thermofisher scientific (SA5-10166) | 1:500 |
| Donkey anti-rabbit Cy3 | Jackson Immuno (711-165-152) | 1:500 |
| Donkey anti-Rabbiy Alexa Fluor 488 | Thermofisher scientific A-21206 | 1:500 |
| EdU Alexa Fluor™ 488 | Thermofisher scientific (C10337) |  |
| Hoechst 33342 | Thermo scientific (62249) | 1:500 |
| **Flow Cytometry** |  |  |
| APC anti-human c-Kit | Biolegend (313206) | 1:100 |
| CXCR4/ CD184 RPE conjugate | Molecular probes (MHCXCR404) | 1:100 |
| **Western Blot** |  |  |
| Anti-APC anti-N- terminal (FE9) | Millipore (OP44) | 1:2000 |
| Anti-APC anti- C-terminal | Abcam (ab154906) | 1:2000 |
