# Supplementary material for: Modeling APC mutagenesis and familial adenomatous polyposis using human iPS cells
Source: PLoS One. 2018 Jul 19;13(7):e0200657. doi: 10.1371/journal.pone.0200657 (PMC6053155; doi:10.1371/journal.pone.0200657)
Supplement: S2 Table — (DOCX) [file pone.0200657.s007.docx]

| **Gene name** | **Probe ID** |
| --- | --- |
| AFP | Hs01040598_m1 |
| AXIN2 | Hs00610344_m1 |
| CDX1 | Hs00156451_m1 |
| CDX2 | Hs01078080_m1 |
| FOXA2 | Hs00232764_m1 |
| GAPDH | [Hs02786624_g1](https://www.thermofisher.com/taqman-gene-expression/product/Hs02786624_g1?CID=&ICID=&subtype=) |
| GATA4 | Hs00171403_m1 |
| LYZ | Hs00426232_m1 |
| MSX1 | Hs00427183_m1 |
| Nanog | Hs02387400_g1 |
| POU5F1 | Hs04260367_gH |
| SOX17 | Hs00751752_s1 |
| T | Hs00610080_m1 |
